# Polybrominated Diphenyl Ethers (PBDEs) in PM_2.5_, PM_10_, TSP and Gas Phase in Office Environment in Shanghai, China: Occurrence and Human Exposure

**DOI:** 10.1371/journal.pone.0119144

**Published:** 2015-03-20

**Authors:** Yue Li, Ling Chen, Duong Minh Ngoc, Yan-Ping Duan, Zhi-Bo Lu, Zhi-Hao Wen, Xiang-Zhou Meng

**Affiliations:** 1 State Key Laboratory of Pollution Control and Resources Reuse, College of Environmental Science and Engineering, Tongji University, Shanghai, China; 2 Shanghai fisheries Research Institute, Shanghai, China; Technion—Israel Institute of Technology, ISRAEL

## Abstract

To evaluate risk via inhalation exposure of polybrominated diphenyl ethers (PBDEs) in office environment, thirty-six pairs air samples including PM_2.5_ (particles with aerodynamic diameter less than 2.5 μm), PM_10_ (particles with aerodynamic diameter less than 10 μm), total suspended particles (TSP) with matching gas phase were collected in office environment in Shanghai, China. The average concentrations of PM_2.5_, PM_10_ and TSP were 20.4, 27.2 and 50.3 μg/m_3_, respectively. Σ_15_PBDEs mean concentrations in PM_2.5_, PM_10_, TSP and gas phase were 51.8, 110.7, 148 and 59.6 pg/m^3^, respectively. Much more PBDEs distributed in fine fractions than coarse ones. PBDEs congener profiles found in PM_2.5_, PM_10_ and TSP (dominated by BDE-209) were different from that in gas phase (dominated by the tri- to penta-BDEs). Approximately 3.20 pg/kg/d PM_2.5_ bound PBDEs can be inhaled into the lung; 3.62 pg/kg/d PM_10_-PM_2.5_(particles with aerodynamic diameter of 2.5-10 μm) bound PBDEs tended to be deposited in the upper part of respiratory system, and the intake of PBDEs via gas-phase was 2.74 pg/kg/d. The exposure of PBDEs was far below the minimal risk levels (MRLs), indicating lower risk from PBDEs via inhalation in the studied office in Shanghai.

## Introduction

In recent years, some studies have shown that some brominated flame retardants, especially polybrominated diphenyl ethers (PBDEs), are potentially toxic, bio-accumulative, ubiquitous and persistent in the environment [1]. Human exposure to PBDEs has been linked to changes in neurodevelopment and reproduction, adverse birth outcomes and disruption of thyroid and reproductive hormone homeostasis [2–4]. As additives, PBDEs can presumably enter the environment by releasing from products, such as televisions, polyurethane, computers, etc [1]. Therefore, the indoor environment has been considered to be important for human exposure to PBDEs. Wu et al.[5] reported that the indoor environment played a prominent role as an exposure media of PBDEs. Many studies have reported PBDEs concentrations in residences in many countries [6–10]. However, information is rather limited regarding levels in offices and other workplaces [11]. Office workers are often in close to office equipment such as computers, printer and other equipment containing PBDEs which may cause serious exposure [12].Therefore, there is a need to assess exposure to PBDEs in office environment.

Like other semi-volatile organic compounds (SVOCs), PBDEs partition between the gaseous and the particulate phase in the atmosphere [13]. These PBDEs bound to particulate matters (PM) may be more persistent in indoor environment due to slow degradation (microbial and photolysis) and other dissipation (volatilization, dissolution) processes [14]. Fine particulates are associated with mortality because they can be deposited much more deeply in the lungs than coarse ones when inhaled. Therefore, indoor PM_2.5_ (particles with aerodynamic diameter less than 2.5 μm) has caused a great deal of attention [15]. Ohura et al. [16] reported that exposure to PM_2.5_ bound PAHs is particularly worrisome for both children and adults who spend over 10 h home every day. Ni et al.[17] measured PBDE levels bound different particle size dust from central air conditioner filters in a new office building. To our best knowledge, no study on PBDEs bound to different size PM in office air has been published.

The objectives of this study were to investigate mass concentrations of PM_2.5_, PM_10_ (particles with aerodynamic diameter less than 10 μm), TSP (total suspended particles) and PBDEs levels in PM_2.5_, PM_10_, TSP and gas phase in a representative office in Shanghai, China; to assess the risk of PBDEs exposure via inhalation in office environment.

## Materials and Methods

### Ethics statement

No specific permissions were required for the described field studies in Shanghai and the field studies did not involve endangered or protected species. The specific location of our study is located at 31°30’N, 121°51’E in Shanghai, China.

### Chemicals

Nineteen PBDEs congeners (BDE-17, 28, 33, 47, 49, 66, 99, 100, 138, 153,154, 183, 190, 196, 203, 206, 207,208, and 209) (purity >99%) were purchased from Accu Standards (New Haven, CT, USA). BDE-50 and BDE-172 used as surrogate standards and BDE-118 (purity >99%) and BDE-128 (purity >99%) used as internal standards were also purchased from Accu Standards.

### Sampling sites

The study was conducted in Shanghai in the Eastern China. The representative office was selected using the following criteria: location, scale, electronic and electrical equipment, flooring, building age and ventilation pattern, *etc*. The selected office building is located in downtown Shanghai which was not adjacent to any industrial activity; it is somehow representative of a white-collar working office. The studied office located on the third floor covers an area of 1450 m^2^ with the height of 3 m. The offices were equipped with common office furniture and electronic products. There is about 350 staff; each of them has a computer and a desktop phone. A printer is shared between 14 staff members. There are also a lot of humidifiers, water dispensers and other electrical and electronic equipment. The office was occupied all the day with a few occupants on duty during the night, so the central air conditioner operated all day. The indoor temperature was 21–25°C and the humility was 45%–70%. The windows of the whole building are completely closed all the time. There are automatic controls for entrance doors of offices. The ventilation rate in this study is significantly lower than others with windows opened (natural ventilation). Central air conditioners are used to recirculate indoor air, with more than 90% of the supply-air typically being recirculated.

### Sample collection

PM_2.5_, PM_10_, TSP and gas phase samples were collected using two high volume air samplers (ECHO HiVol, Italy).Six sampling events were conducted in June, July, August, December 2012 and January, February 2013, respectively. During sampling, particles and gas phase samples were isolated from the atmosphere by drawing air through a quartz fiber filter (QFF, 102 mm in diameter, Munktell, Sweden) and polyurethane foam (PUF, 65 mm× 75 mm, TISCH, USA)at a flow rate of about 0.2 m^3^/min. PM_2.5_ and PM_10_ were collected using PM_2.5_ and PM_10_ cyclones, respectively. To avoid disturbing most staff during the day, the sampler was operated from midnight to the early morning and the sampling process was repeated for 3 days until 300 m^3^ of air was sampled. The time interval for collecting PM_2.5_, PM_10_ and TSP with matching gas phase samples was 7 days. To obtain duplicate samples, two air samplers were operated simultaneously. All samples were collected from the atmosphere at a height of 1.5 m above ground.

Before being used, QFFs were baked at 450°C for 4 h and PUF plugs were Soxhlet extracted for 48 h with methanol and for another 48 h with an acetone and hexane mixture (1:1) to remove any organic containment. After sampling, the QFFs were wrapped with aluminum foils and sealed in double-layer polyethylene bags, and PUFs were placed in solvent rinsed glass cartridges with PTEE covers, and then transported to the laboratory and stored at -20°C until extraction. The concentrations of PM_2.5_, PM_10_ and TSP were determined by weighing the filters after conditioning the filter at 33% relative humidity and 22°C before and after sampling.

### Sample extraction and PBDEs analysis

The QFFs and PUF plugs spiked with surrogate standards were extracted with a Soxhlet extractor using 150 ml dichloromethane for 48 h. The extract was rotary-evaporated to about 2 ml and solvent-exchanged to hexane, and then 4 ml concentrated sulfuric acid was added. Each extract was subsequently cleaned with a gel permeation chromatography column (40 g Bio-Beads SX-3). The final extract volume was evaporated to 100 μL under a gentle N_2_ stream. Internal standards were added before instrumental analysis.

PBDEs congeners were quantified with a Shimadzu GCMS-QP 2010 plus instrument with the selective ion monitoring (SIM) mode. A DB-5 column (15 m × 0.25 mm × 0.1 μm, J&W Scientific) was employed. The analysis of PBDEs congeners using the isotope internal standard method followed our previous study[18].

### Quality assurance/quality control (QA/QC)

Breakthrough of gas-phase chemicals was tested by using a second PUF plug in series with the first one. The PBDEs contents in the second plug were less than 7% of those in the first plug for all congeners.

For each batch of PM_2.5_, PM_10_, TSP and gas samples, a procedural blank, spiked blank and drift tests were processed. All targets were lower than the limit of detection (LOD) in procedural blanks, so they were not subtracted from the sample measurement. The surrogate recovery of BDE-50 and BDE-172 was 79.3 ± 4.5% and 86.5 ± 6.5%, respectively. The quantification was performed with a seven-point calibration curve with 20 μg/L ^13^C-BDE-118 and ^13^C-BDE-128 as internal standards (*R* ^*2*^ >0.999) for both lower and higher BDE congeners. Both external calibration and internal standard (BDE-118) calibration were applied in the quantification of BDE-209, while internal standard calibration was used for other congeners. To check drift, standards were run after every 6 samples, and the results were unacceptable unless the relative standard deviation for individual PBDEs congener in standards was less than 10%. Recoveries of targets ranged from 81.5% to 89.7%, and relative standard deviations ranged from 3.2% to 7.9% in spiked blank samples. Reported concentrations were not surrogate recovery corrected.

### Risk assessment

Based on the NOAEL_HEC_ values (human equivalent no-observed-adverse-effect level), the MRL (Minimal Risk Levels) is derived as follows:

$$MRL=(NOAEL_{HEC})÷(\text{UF}\times \text{MF})$$ (1)

where UF is uncertainty factor and MF is modifying factor. This method is limited to intermediate-duration (15–364 days) inhalation study of PBDEs.

The NOAEL_HEC_ was calculated from the duration-adjusted NOAEL (NOAEL_ADJ_) using EPA RfDs methodology as follows:

$$NOAEL_{HEC}=NOAEL_{ADJ}\times RDDR$$ (2)

The regional deposited dose ratio (RDDR) (2.7) for the extrathoracic (ET) region was used to extrapolate deposited doses in species studied to deposited doses in human [19].

For the lower brominated PBDEs: NOAEL was 1.1 mg/m^3^;

$$NOAEL_{ADJ}=1.1mg/m^{3}\times 6\;hours/24hours\;\times 5days/7days=0.196mg/m^{3}$$

$$NOAEL_{HEC}=0.196mg/m^{3}\times 2.7=0.53mg/m^{3}$$

The MRL was derived by dividing the NOAEL_HEC_ by UF of 30 (3 for species to species extrapolation with dosimetric adjustments and 10 for human variability) and MF of 3 (for an incomplete database reflecting a single study in one species).

$$MRL=0.53\;mg/m^{3}÷(30\times 3)=0.006mg/m^{3}$$

For higher brominated PBDEs: The MRL was estimated by dividing the NOAEL (1000 mg/kg/day) by UF of 100 (10 for animal to human extrapolation and 10 for human variability). It has been suggested that deca-BDE is very poorly absorbed via inhalation (≈ 1% or less of an oral dose) and rapidly eliminated (≈ 99% of the dose within 72 hours) [20]. Then 0.001 mg/kg/day for inhalation exposure was derived. For adults, the inhalation value of 0.07 mg/day was estimated by multiplying by the body weight (70 kg) [21]. Considering the inhalation rate (0.54 m^3^/h = 12.96 m^3^/day [21]), the final MRL for deca-BDE was 0.005 mg/m^3^.

### Statistical Analysis

One-way ANOVA was performed to assess the significance of differences. Statistical significance was evaluated at *p*<0.05 level. The measured PBDE data followed the normal distribution with the average and standard deviations. Data were not transformed during the analysis. All statistical analyses were performed with SPSS software (Ver 13.0; SPSS, Chicago, IL, USA).The experimental data were expressed as mean ± standard deviation (SD).

## Results and Discussion

### Average concentrations of PM_2.5_, PM_10_ and TSP

Because there was no significant difference between the mass concentrations of suspended particles in summer and winter in the office the average concentrations of these particles were applied (S1 Table). Table 1 showed the concentrations of PM_2.5_, PM_10_ and TSP in this study and previous studies. The average concentrations of PM_2.5_, PM_10_ and TSP were 20.4, 27.2 and 50.3 μg/m^3^, respectively. Higher concentrations of indoor PM were generally found in developing countries than developed countries [22]. The office concentration of PM_2.5_ in this study was comparable to those reported in offices in Beijing (China) [23], but much lower than those from Belgium [24]and Greece [22, 25]. Compared with the Global Air Quality Guidelines set by World Health Organization (10 μg/m^3^ for annual mean)and U.S. annual health standard (12 μg/m^3^)[26], the PM_2.5_ level in this study was slightly higher. In the European Union, the Air Quality Directive (Directive 1999/30/EC) established the limit values for PM_10_, to be accomplished by 2010 (20 μg/m^3^ as annual daily value)[27]. The concentration of PM_10_ in this study was higher than this directive. Those regulations were established for ambient air pollution. It is inappropriate to compare the indoor measurements with them, however, there is no any other regulations for indoor air quality. The ambient standard is considered to be a reasonably conservative benchmark for occupational exposure since it represents an effective maximum allowable concentration for the general population. The indoor air quality regulation is still required, so further work is needed to do to get more information to support more definitive occupational exposure.

**Table 1:** Average concentrations of PM_**2.5**_, PM_**10**_ and TSP in the present study and those from previous studies (μg/m^**3**^).

Location	Type	PM_2.5_	PM_10_	TSP	PM_2.5_/PM_10_	PM_2.5_/TSP	PM_10_/TSP	ETS	Ref
Beijing, China	Offices	28.1	63	105	0.45	0.27	0.6	No	[23]
Bhilai-Durg, India	Offices	-	278.8±84.3	-	-	-	-	Yes	[28]
Antwerp, Belgium	Offices	59±7	67±8	-	0.88	-	-	Yes	[24]
Antwerp, Belgium	Offices	15±0.9	20±1	-	0.75	-	-	No	[24]
Athens, Greece	Offices	199	281	-	0.71	-	-	Yes	[25]
Seoul, Korea	Offices	-	99±68	-	-	-	-	Yes	[29]
New, Jersey, USA	Offices	-	30.3±17.6	-	-	-	-	No	[30]
Paris, France	Offices	-	53	-	-	-	-	No	[31]
Thessaloniki, Greece	Offices	67±32	93±43	-	0.72	-	-	No	[22]
Shanghai, China	Offices	20.4±6.9	27.2±9.3	50.3±15.7	0.75	0.40	0.54	No	This study

ETS, represents environmental tobacco smoking.

Except for the offices in Belgium, the concentrations in this study were obviously lower compared to others [22–25, 28–31]. Several factors influenced the concentrations of PM_2.5_, PM_10_, and TSP in indoor air. It is generally thought that indoor concentrations of particles derive from indoor and outdoor sources, determined by several variables: air-exchange rate, outdoor air quality, indoor activities, aerodynamic diameter of PM emitted. etc. [32]. Because the central air conditioner operated all day, the air exchange rate (0.5 h^-1^)in this study is significantly lower than others with windows opened (natural ventilation)[11]. The filtration units installed with the air conditioners can also remove some of the particulate. This makes the indoor and outdoor correlation poor in the investigated office. Therefore, indoor activities and aerodynamic diameter of PM emitted may be the principal factors, especially, the tobacco smoking[33]. As expected, the smokers' office showed higher indoor PM concentrations than without environmental tobacco smoking in Table 1. Smoking is prohibited in the entire building in this study, which might be one of the reasons that the concentrations of particles were lower than others. Researchers also found that occupant-related activities may also be the source (composed of cloth fibers, skin cells, resuspended particles of origin by walking, and emission from materials handled) of the indoors with no specific source (like smoking and combustion of fuel) [34].

The ratio of PM_2.5_/PM_10_ mass concentrations was calculated as presented in Table 1. The PM_2.5_/PM_10_ ratio in the studied office was significantly higher than previous studies [23, 35]. A PM_2.5_/PM_10_ ratio of 0.5 is typical of developing country urban areas and is at the bottom of the range found in developed country urban areas (0.5–0.8) [36]. The result reflected that Shanghai was in the similar situation with developed countries. Higher PM_2.5_ /PM_10_ ratio may also be explained by the release of ultrafine particles from office equipment such as printers and copiers. There were about twenty-six printers and copiers in the studied office. Reports have identified ultrafine aerosol particle formation during operation of laser printer and copiers [37, 38]. In the study of Koivisto et al. [39], they presented how an indoor aerosol model can be used to characterize particle emitter and predict influence of the source on indoor air quality. According to their model, peak emission rates of the printers exceeded 7.0×10^8^ s^-1^, and emitted mainly ultrafine particles (diameter less than 100 nm). Printer-emitted particles increased 6-h averaged particle concentration over eleven times compared to the background particle concentration. Toner and paper dust from printing devices may become airborne, generating respirable particles (PM_2.5_) [38, 40].

### PBDEs concentrations and profiles

**Fig 1:**
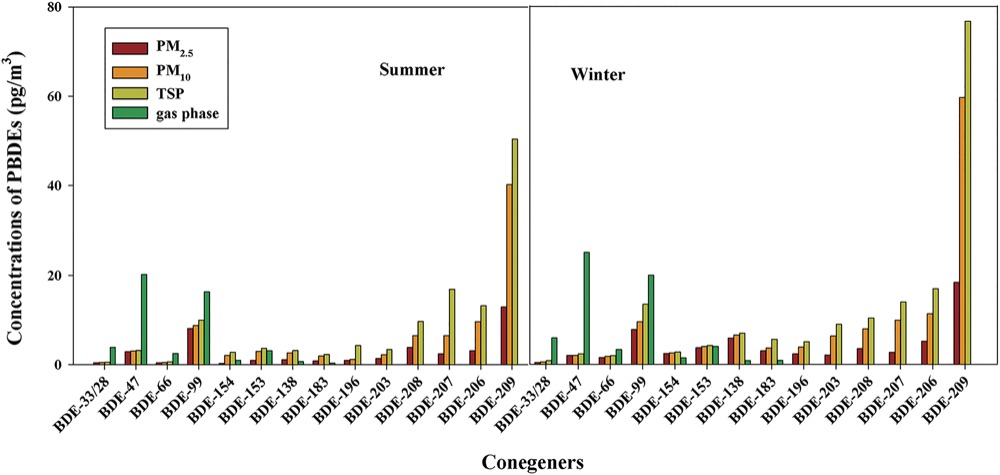
Concentrations of PBDEs congeners in PM_2.5_, PM_10_, TSP and gas phase (Summer: June, July and August 2012; Winter: December 2012, January, and February 2013).

Among 19 target PBDE congeners, only those detected in at least 60% of the samples were quantified, including BDE-28, 33, 47, 66, 99,138, 153, 154, 183, 190, 196,203, 206,207 and 209. Here we defined Σ_15_PBDEs as the sum of all these congeners. Concentrations of PBDEs in PM_2.5_, PM_10_, TSP and gas phase in summer and winter are presented in Fig. 1 and S2–S7 Table. The PBDEs congeners profile did not show notable seasonal variations (P>0.05). The office was occupied around the clock with a few occupants on duty during the night. The central air conditioner operated all day, so the temperature and humidity of the room fluctuated in a narrow range. The indoor temperature was 21–25°C and the humility was 45%–70%. Moreover, sample sizes were not large enough to cover all the differences statistically. Nevertheless, the concentrations in winter were slightly higher than those in summer. This may be caused by the higher organic carbon of the particulate in winter. Baek et al [29] investigated the seasonality on indoor air quality (homes, offices), and there was a general pattern of increasing levels from summer to winter for RSP and volatile organic compounds. Han et al. [41] found stronger correlation between PBDEs with organic carbon of particles suggesting that part of PBDEs was absorbed into the organic matter. This slight difference may also be caused by the smaller sample size.

Octa-, nona- and deca-BDE were only found in the particulate phase, whereas the tri-BDE (BDE-28/33) and BDE-47 were almost exclusively present in the gas phase (89–93% and 86–91%). The tetra- to hepta-BDEs were both found in the particulate and gas phase. PBDEs distributed disproportionally between the fine and coarse fractions. Much more PBDEs distributed in fine fractions than coarse ones, especially for lower brominated PBDEs. Fine fractions contain more PBDEs, indicating higher accumulative effect. This was consistent with the result found by Wang et al. [8]; more than 80% of ΣPBDEs were associated with fine particles implying a definite enrichment in fine particles. Meanwhile, over 46% ΣPBDEs in atmospheric particles of Greece [42] were associated with particles <0.57 μm in diameter, and PBDEs in particles of larger size were substantially lower. However, the average PM_2.5_/TSP ratio of BDE-209 concentration was 26% in winter and 24% in summer suggesting higher brominated PBDEs existed mainly in coarse particles. BDE-209 concentrations in particles 2.5–10 μm in diameter were 64% and 54% in summer and winter, respectively. It was also found that BDE-209 was mainly associated with particles 3–10 μm in diameter [43].

No publication data on PBDEs concentrations in PM_2.5_ and PM_10_ in office air were available. PBDEs bound to different size of dust in the central air conditioner filter of an office building in Shenzhen, China [17]were selected for comparison, because central air conditioner filter dust was able to cover the essential information of PBDEs in indoor environments in a given time interval. As shown in Table 2, PBDEs concentrations in PM_2.5_ and PM_10_ in this study were slightly higher than that in dust in conditioner filter due to the different sample types and sources[17].

**Table 2:** Comparison of PBDEs concentrations in PM_**2.5**_, PM_**10**_, TSP and gas phase in office environment (pg/m^**3**^).

	Location	Number	BDE-28/33	BDE-47	BDE-99	BDE-153	BDE-209	Σ_15_PBDEs	Ref
PM_2.5_	Shanghai, China	6 (summer)	0.45	2.89	8.10	1.01	12.9	41.1	This study
	Shanghai, China	6 (winter)	0.39	1.98	7.81	3.69	18.3	62.5	This study
	Shenzhen, China^a^	56	0.014	0.021	0.014	0.004	7.285	10.84	[17]
PM_10_	Shanghai, China	6 (summer)	0.49	3.01	8.75	2.98	45.3	89.3	This study
	Shanghai, China	6 (winter)	0.53	2.01	9.52	3.98	59.5	132	This study
	Shenzhen, China^a^	56	0.089	0.132	0.086	0.022	44.75	66.61	[17]
TSP	Shanghai, China	6 (summer)	0.53	3.22	9.98	3.69	50.4	126	This study
	Shanghai, China	6 (winter)	0.79	2.32	13.4	4.32	70.5	170	This study
	Michigan, America^b^	13	8	933	156	-	-	1170	[11]
	Sweden^c^	6	-	760	380	22	170	-	[44]
Gas	Shanghai, China	18 (summer)	3.98	20.2	16.3	3.11	-	52.8	This study
	Shanghai, China	18 (winter)	5.91	25.3	19.9	3.96	-	66.4	This study
	Michigan, America^b^	18	404	1490	259	-	-	3400	[11]
	Boston, America^d^	31	57	263	82	5.8	-	-	[45]
	Greece^e^	5	-	-	-	-	-	205	[46]
	Kuwait^f^	24	0.8	19.0	6.7	0.2	-	32.7	[47]

^a^ central air conditioner filter dust samples, the sum of BDE-28, 47, 49, 85, 99, 100, 153, 154, 138, 183, 196, 206, 207, 208, 209;

^b^ the sum ofBDE-17, 28, 75, 49, 71, 47, 66, 100, 99, 85, 154, 153, 138, 166, 183, 190, 203, 208, 207, 206, 209;

^c^ the sum of BDE-47, 100, 99, 85, 154, 153, 128, 183, 209;

^d^the sum of BDE-28, 47, 49,66, 75, 85, 99, 100, 138, 153, 154, 183;

^e^ the sum of BDE-15, 32, 36, 17 25, 39, 38, 35, 62, 49, 47, 66, 100, 99, 154, 153, 183;

^f^ the sum of 1, 2, 3, 7, 8, 10, 11, 12, 13, 15, 17, 25, 28, 30, 32, 33, 35, 37, 47, 49, 66, 71, 75, 77, 85, 99, 100, 116, 118, 119, 126, 138, 153, 154, 155, 166, 181, 183, 190,209.

Concentrations of Σ_15_PBDEs in TSP (124, 170 pg/m^3^ for summer and winter) were 1 order of magnitude lower than America [11] and Sweden [44]. BDE-209 was the dominant congener suggesting that commercial deca-BDE may have been added into the appliances. This was consistent with other studies in China [48, 49]. BDE-209 accounted for 40% and 45% of total PBDEs in summer and winter, respectively. These values were lower than the other air studies [50, 51]. The nona-BDE congeners constituted were higher than what was reported in the deca-BDE commercial mixture [52]. This may be due to debromination of BDE-209 in the atmosphere. However, higher nona-BDE may be derived from the octa-BDE DE-79 and Bromkal 79–8DE [52]. The Σ_15_PBDEs in gas phase (52.1 pg/m^3^, 66.4 pg/m^3^for summer and winter) in this study were lower than those reported in Greece [46], and America [11], but slightly higher than Kuwait [47]. This may be due to the room characteristics and potentially flame-retarded items. However, this analysis cannot be conducted appropriately in this study unless detailed information about different offices was obtained. Further study about the correlation between different PBDEs levels and room characteristics is required. The average ratio of BDE-47 to BDE-99 in air (including TSP and gas phase) were 1.17, between those in the technical penta-BDE mixture Bromkal 70–5DE (1.05) and DE-71 (1.27) [52], suggesting that a penta-BDE commercial mixture may also been the formula used in the equipment of the office.

### Risk assessment

#### Daily exposure to PBDEs via inhalation

The office exposure can occur from air inhalation, dust ingestion and dermal contact. The exposure was assessed only via inhalation pathways to address inhalation exposure in office environment [21]. In this study, the risk of potential adverse health effects was calculated using mean concentrations of PBDEs in PM_2.5_, PM_10_, TSP and the gas phase during summer and winter. The exposed persons were all adults. The daily exposure dose by air inhalation (DED_air_) of PBDEs in gas phase can be estimated as:

DEDair=C×IR×T×frbw (3)

where C is the average PBDEs concentration in gas phase (pg/m^3^), IR is inhalation rate (m^3^/d), T is exposure time (h), f_r_ is the alveolar fraction retained in the lungs (a value of 0.75 was used [53]) and bw is the body weight of the exposed person (kg). With IR = 0.54 m^3^/h, bw = 70 kg (adults)[21], T is the exposure duration (8 h per day in the studied office).

The daily exposure dose by air inhalation (DED_air_) of Σ_15_PBDEs in different sizes of particulate matter was estimated using the following equation.

DEDair=CPM×IR×Tbw (4)

Where C_PM_ (pg/m^3^) is the PBDEs concentration in PM. Human exposure via inhalation of PM-bound and gas-phase PBDEs were calculated and presented in Table 3.

**Table 3:** Exposures of BDEs of PM-bound and gas-phase via inhalation (pg/kg/d).

	BDE-33	BDE-47	BDE-100	BDE-99	BDE-153	BDE-138	BDE-209	Σ_15_PBDEs
PM_2.5_	0.03	0.15	0.05	0.49	0.15	0.22	1.18	3.20
PM_10_	0.03	0.16	0.06	0.56	0.22	0.28	3.08	6.82
PM_10_-PM_2.5_	0.01	0.01	0.01	0.07	0.07	0.07	2.11	3.62
TSP	0.04	0.17	0.10	0.72	0.24	0.31	3.92	9.05
gas	0.23	1.05	0.13	0.84	0.16	0.04	-	2.74
Total	0.27	1.22	0.23	1.56	0.41	0.35	3.92	11.8

**Fig 2:**
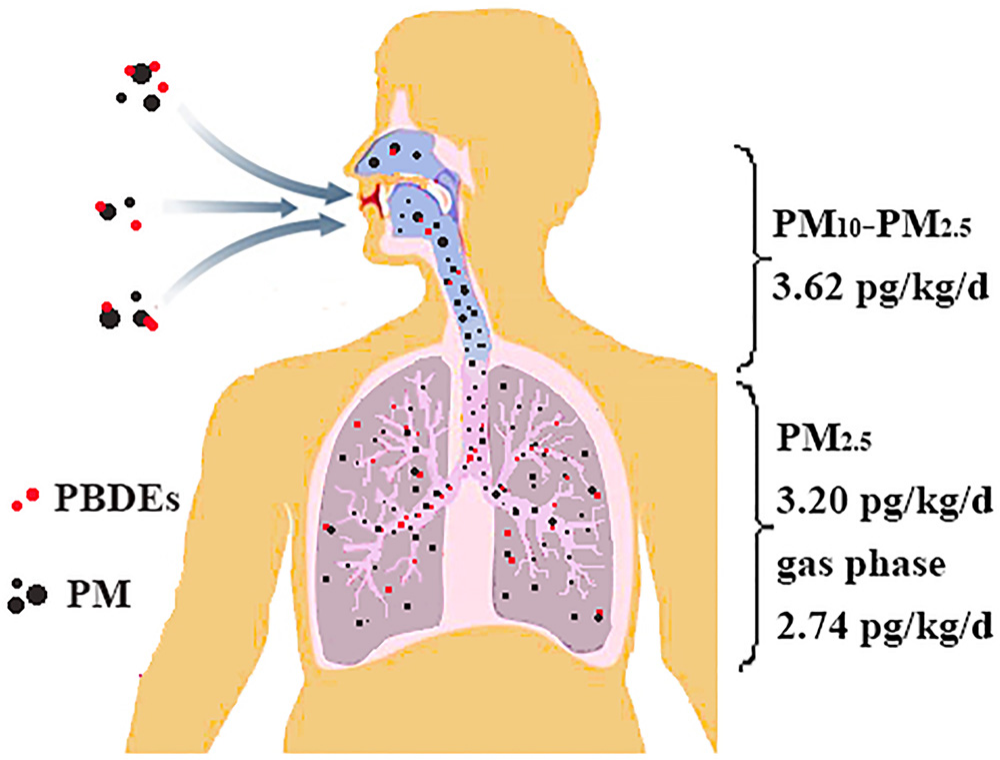
Daily exposure to PBDEs via inhalation of PM_2.5_, PM_10_, TSP and gas phase.

As shown in Fig. 2, about 3.20(0.69–4.33) pg/kg/d PM_2.5_ bound PBDEs can be inhaled deep into the lungs and reach the pulmonary alveoli. They can cause breathing and respiratory problems [15]. The values were much higher than that (0.67 pg/kg /d) obtained via dust of central air conditioner filter with particulate diameter of 0.4–2.2 μm [17]. Meanwhile, 3.62 pg/kg/d PM_10_-PM_2.5_ bound PBDEs tended to be deposited in the upper parts of the respiratory system. However, this part of PBDEs can generally be expelled back into the throat and therefore do less harm to human health than PM_2.5_. The mean concentrations of PBDEs bound to particulates with diameter greater than 10 μm were 2.23 pg/kg/d. Due to inhalable particles excluding these particulates, the potential harm of this portion can be ignored in consideration of respiratory exposure. However, this portion can also be cleared from the extrathoracic region and the tracheobronchial compartment via mucociliary clearance and ingested. So this part should be taken into consideration when estimate the accurate ingestion exposure. Approximately 2.74 pg/kg/d PBDEs can be inhaled in the lungs via gas-phase. The total intake of PBDEs (11.8 pg/kg/d) was far below the lowest observed adverse effect level of 1 mg/kg/d recommended by Darnerud et al.[54]. The USEPA recommended the RfD of daily oral PBDEs exposure, and the RfD values were 0.1, 0.1, 0.2 and 7 μg/kg/d for BDE-47, 99, 153 and 209, respectively [55]. Obviously, the average intake of PBDEs congeners via inhalation for occupants in this office was far below the recommended RfD values. However, other studies have suggested that inhalation is only a minor exposure route compared to incidental dust ingestion and diet [9]. Therefore, overall and accurate risk assessment in workplace is necessary in the further work (i.e. the exposure associated with dust).

#### Health risks of inhalation PBDEs exposure

**Fig 3:**
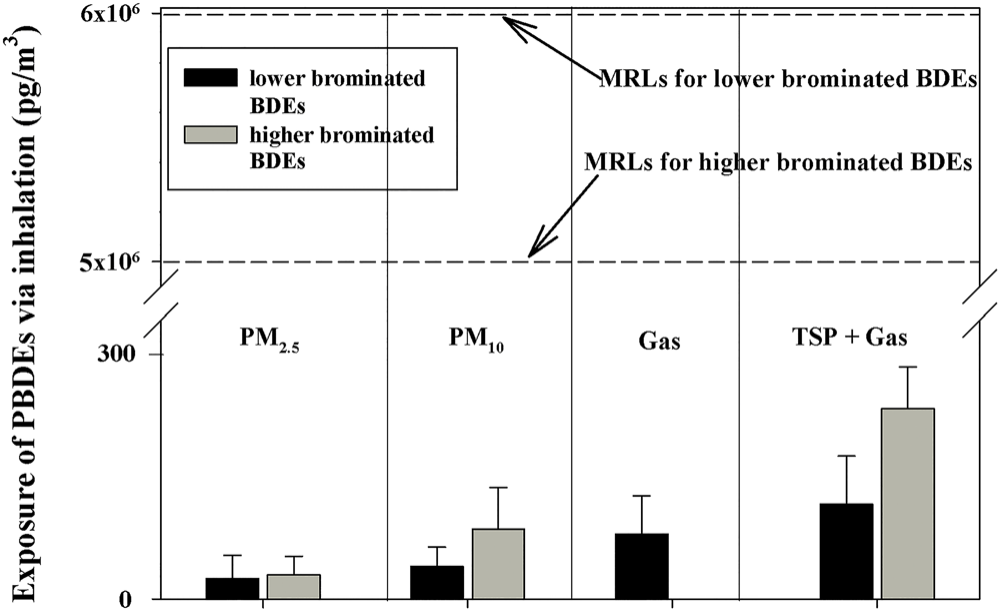
Health risks of inhalation PBDEs exposure via PM_2.5_, PM_10_, TSP and gas phase.

People are exposed to environmental PBDE mixtures of different congeners rather than the original commercial PBDE products. However, the toxicity data based on MRLs for environmental PBDEs are insufficient. Hence data from commercial PBDE mixtures are used to develop MRLs from environmental concentrations to PBDEs, owing to the following factors: (1) multiple mechanisms may be involved in health effects induced by PBDEs; (2) different PBDE congeners may produce effects by different mechanisms; (3) humans are exposed to complex mixtures of interacting PBDEs with differing biological activities, as well as to the lack of a suitable approach for quantitatively evaluating joint toxic action from concurrent exposures to PBDEs in the environment [56]. An MRL of 0.006 mg/m^3^ has been derived for intermediate-duration inhalation exposure (15–364 days) to lower brominated BDEs [57]. (The calculation of MRL was presented in materials and methods) There are obvious differences in the environmental chemistry and toxicity of deca-BDE compared to lower brominated BDEs. However, no MRL was derived for acute-, intermediate-, or chronic-duration inhalation exposure to deca-BDE due to a lack of inhalation studies for this PBDE congener. It has been suggested that deca-BDE is very poorly absorbed via inhalation (≈ 1% or less of an oral dose) and rapidly eliminated (≈ 99% of the dose within 72 hours) [20]. Therefore, inhalation MRL of deca-BDE in this study is derived based on the study of oral exposure to deca-BDE [20]. An MRL of 0.005 mg/m^3^ was derived for intermediate-duration inhalation exposure to deca-BDE. We divided the targets 15 PBDE congeners into two categories: the lower brominated BDEs (including BDE-28, 33, 47, 66, 99, 138, 153, 154, and 183) and higher brominated BDEs (including BDE-196, 203,206, 207, 208 and 209). As shown in Fig. 3, exposure to higher brominated BDEs via inhalation was only contributed by PM. The values were far below the MRLs (0.005 mg/m^3^), indicating much lower risk for higher brominated BDE in the surrounding air. Exposure to lower brominated BDEs was 22.32, 35.79 and 70.98pg/m^3^ for PM_2.5_, PM_10_ and gas phase respectively. The exposure to lower brominated BDEs did not exceed the value of MRLs (0.006 mg/m^3^), indicating lower risk of adverse health effects for inhalation exposure during working time. The present study provides useful information about PBDEs exposure and the risk of adverse health effects for office occupation population during working time. However, the MRLs in this study were derived for intermediate-duration (15–364 days) inhalation exposure. No MRLs were derived for chronic-duration inhalation exposure to PBDEs due to a lack of inhalation studies. Further study on chronic-duration inhalation exposure to PBDEs was required.

## Conclusions

This study was the first in Shanghai, China to address exposure to PBDEs via PM_2.5_, PM_10_, TSP and gas phase in office environment. Both mass concentrations of PM_10_ and PM_2.5_were higher than the European Air Quality Directive, Global Air Quality Guidelines set by World Health Organization and U.S. annual health standard, suggesting higher PM_2.5_ exposure in the studied office. The PBDE congeners profile did not show notable seasonal variation. PBDEs were primarily associated with the fine fractions compared to coarse ones, especially for lower brominated PBDEs. PBDEs exposure via inhalation was below the RfD values recommended by USEPA. Both exposure amount of lower and higher brominated BDEs in PM_2.5_, PM_10_ and gas phase were far below their MRLs values, which were 0.006 mg/m^3^ for lower brominated BDEs and 0.005 mg/m^3^ for higher brominated BDEs, indicating lower risk for PBDEs in the office environment. However, further work of PBDEs exposure for office workers was also required to allow for overall and accurate risk assessment.

## Supporting Information

S1 Table Particulate mass concentrations (μg/m^3^) for PM_2.5_, PM_10_ and TSP. (DOCX)

S2 Table PBDEs concentrations (pg/m^3^) in different particulate matter and gas phase in June, 2012. (DOCX)

S3 Table PBDEs concentrations (pg/m^3^) in different particulate matter and gas phase in July, 2012. (DOCX)

S4 Table PBDEs concentrations (pg/m^3^) in different particulate matter and gas phase in August, 2012. (DOCX)

S5 Table PBDEs concentrations (pg/m^3^) in different particulate matter and gas phase in December, 2012. (DOCX)

S6 Table PBDEs concentrations (pg/m^3^) in different particulate matter and gas phase in January, 2013. (DOCX)

S7 Table PBDEs concentrations (pg/m^3^) in different particulate matter and gas phase in February, 2013 (DOCX)
