# Supplementary material for: Polybrominated Diphenyl Ethers (PBDEs) in PM2.5, PM10, TSP and Gas Phase in Office Environment in Shanghai, China: Occurrence and Human Exposure
Source: PLoS One. 2015 Mar 20;10(3):e0119144. doi: 10.1371/journal.pone.0119144 (PMC4367993; doi:10.1371/journal.pone.0119144)
Supplement: S1 Table — (DOCX) [file pone.0119144.s001.docx]

Table S1. Particulate mass concentrations (μg/m^3^) for PM_2.5_, PM_10_ and TSP.

|  | Jun, 2012 | | Jul, 2012 | | Aug, 2012 | | Dec, 2012 | | Jan, 2013 | | Feb, 2013 | |
| --- | --- | --- | --- | --- | --- | --- | --- | --- | --- | --- | --- | --- |
| PM_2.5_ | 18.5 | 13.2 | 15.2 | 14.3 | 20.3 | 18.4 | 24.3 | 28.3 | 29.6 | 21.3 | 19.3 | 26.2 |
| PM_10_ | 24.9 | 20.3 | 19.5 | 26.1 | 35.1 | 24.6 | 20.3 | 26.7 | 25.9 | 32.1 | 42.9 | 48.3 |
| TSP | 41.5 | 35.2 | 65.3 | 58.4 | 45.6 | 55.7 | 75.9 | 68.2 | 37.6 | 35.9 | 40.3 | 33.8 |
