# Supplementary material for: Polybrominated Diphenyl Ethers (PBDEs) in PM2.5, PM10, TSP and Gas Phase in Office Environment in Shanghai, China: Occurrence and Human Exposure
Source: PLoS One. 2015 Mar 20;10(3):e0119144. doi: 10.1371/journal.pone.0119144 (PMC4367993; doi:10.1371/journal.pone.0119144)
Supplement: S2 Table — (DOCX) [file pone.0119144.s002.docx]

Table S2. PBDEs concentrations (pg/m^3^) in different particulate matter and gas phase in June, 2012.

|  | PM_2.5_ | gas | PM_2.5_ | gas | PM_10_ | gas | PM_10_ | gas | TSP | gas | TSP | gas |
| --- | --- | --- | --- | --- | --- | --- | --- | --- | --- | --- | --- | --- |
| BDE-28/33 | 0.45 | 3.91 | 0.53 | 2.54 | 0.49 | 5.21 | - | 3.69 | 0.53 | 4.52 | - | 7.52 |
| BDE-49 | 0.29 | 1.95 | - | 2.24 | 0.31 | 2.65 | 0.12 | 1.98 | 0.36 | 2.32 | - | 2.65 |
| BDE-47 | 2.89 | 20.2 | 1.23 | 25.3 | 3.01 | 32.2 | 3.21 | 28.8 | 3.29 | 15.6 | 3.85 | 29.9 |
| BDE-66 | 0.45 | 2.54 | 0.98 | - | 0.51 | 1.99 | - | 2.56 | 0.65 | 0.74 | 0.59 | 1.98 |
| BDE-100 | 0.25 | 2.13 | 0.54 | 0.98 | 0.3 | 1.65 | 0.97 | 3.24 | 0.36 | 0.99 | 1.52 | 1.11 |
| BDE-99 | 8.11 | 16.3 | 6.31 | 10.3 | 8.75 | 27.7 | 6.31 | 19.7 | 9.98 | 25.7 | 6.31 | 23.4 |
| BDE-154 | 0.27 | 0.97 | 0.35 | 0.12 | 2.07 | 0.24 | 1.21 | 0.87 | 2.74 | 0.54 | 3.52 | 0.68 |
| BDE-153 | 1.01 | 3.1 | 1.98 | 0.25 | 2.98 | 0.74 | 1.32 | 0.99 | 3.69 | 1.87 | 5.02 | 2.54 |
| BDE-138 | 1.12 | 0.68 | 1.65 | - | 2.64 | 0.03 | 1.97 | - | 3.21 | 1.51 | 2.34 | 2.66 |
| BDE-183 | 0.85 | 0.35 | 1.21 | - | 1.91 | - | 1.05 | - | 2.65 | - | 4.25 | 1.87 |
| BDE-196 | 0.97 | - | 0.85 | - | 1.18 | - | 1.03 | - | 4.25 | - | 3.21 | - |
| BDE-203 | 1.37 | - | 1.59 | - | 2.25 | - | 2.15 | - | 3.39 | - | 3.64 | - |
| BDE-208 | 3.87 | - | 2.98 | - | 6.52 | - | 4.59 | - | 9.68 | - | 8.65 | - |
| BDE-207 | 2.39 | - | 2.14 | - | 6.51 | - | 4.39 | - | 16.9 | - | 22.6 | - |
| BDE-206 | 3.12 | - | 3.09 | - | 9.65 | - | 5.34 | - | 13.2 | - | 10.3 | - |
| BDE-209 | 12.9 | - | 19.7 | - | 40.2 | - | 30.2 | - | 50.4 | - | 70.9 | - |
