# Supplementary material for: Polybrominated Diphenyl Ethers (PBDEs) in PM2.5, PM10, TSP and Gas Phase in Office Environment in Shanghai, China: Occurrence and Human Exposure
Source: PLoS One. 2015 Mar 20;10(3):e0119144. doi: 10.1371/journal.pone.0119144 (PMC4367993; doi:10.1371/journal.pone.0119144)
Supplement: S3 Table — (DOCX) [file pone.0119144.s003.docx]

Table S3. PBDEs concentrations (pg/m^3^) in different particulate matter and gas phase in July, 2012

|  | PM_2.5_ | gas | PM_2.5_ | gas | PM_10_ | gas | PM_10_ | gas | TSP | gas | TSP | gas |
| --- | --- | --- | --- | --- | --- | --- | --- | --- | --- | --- | --- | --- |
| BDE-28/33 | 0.65 | 2.65 | - | 2.09 | - | 5.21 | - | 3.05 | - | 8.36 | 0.21 | 5.47 |
| BDE-49 | 0.13 | 3.14 | - | 1.25 | - | 2.19 | 0.21 | 3.27 | 0.12 | 0.54 | - | 0.65 |
| BDE-47 | 0.89 | 25.6 | 2.85 | 26.7 | 2.18 | 29.7 | 3.54 | 25.8 | 3.14 | 12.5 | 4.1 | 35.1 |
| BDE-66 | 0.76 | 3.25 | 1.11 | 0.88 | - | 2.25 | - | 5.97 | 0.48 | 0.68 | 0.75 | 1.13 |
| BDE-100 | 0.98 | 1.05 | 0.72 | 3.25 | 0.87 | 3.41 | 0.24 | 4.38 | 0.97 | 2.98 | 0.41 | 4.58 |
| BDE-99 | 5.74 | 9.13 | 6.78 | 7.51 | 5.91 | 21.6 | 5.74 | 19.7 | 5.21 | 10.3 | 8.41 | 27.9 |
| BDE-154 | - | 1.98 | 0.89 | 2.11 | 1.52 | 0.66 | 0.94 | 1.54 | 1.99 | 0.14 | 2.65 | 0.54 |
| BDE-153 | 0.99 | 1.68 | 1.98 | 3.55 | 1.58 | 2.99 | 2.32 | 8.14 | 4.97 | 6.84 | 4.97 | 4.31 |
| BDE-138 | 1.95 | 0.41 | 1.56 | 0.24 | 2.58 | 0.04 | 2.19 | 0.12 | 3.68 | 0.87 | 4.98 | 1.55 |
| BDE-183 | 1.52 | 0.97 | 0.87 | - | 1.35 | - | 2.15 | 1.34 | 2.98 | 0.11 | 1.97 | - |
| BDE-196 | 0.75 | - | 1.21 | - | 1.65 | - | 1.97 | - | 2.56 | - | 3.07 | - |
| BDE-203 | 1.28 | - | 1.02 | - | 1.54 | - | 1.74 | - | 4.06 | - | 5.14 | - |
| BDE-208 | 2.59 | - | 3.21 | - | 5.13 | - | 8.51 | - | 6.78 | - | 5.78 | - |
| BDE-207 | 2.01 | - | 1.98 | - | 3.97 | - | 7.21 | - | 18.5 | - | 19.6 | - |
| BDE-206 | 2.65 | - | 2.48 | - | 4.39 | - | 8.75 | - | 11.5 | - | 13.8 | - |
| BDE-209 | 8.32 | - | 10.2 | - | 39.2 | - | 37.2 | - | 40.3 | - | 60.3 | - |
