# Supplementary material for: Polybrominated Diphenyl Ethers (PBDEs) in PM2.5, PM10, TSP and Gas Phase in Office Environment in Shanghai, China: Occurrence and Human Exposure
Source: PLoS One. 2015 Mar 20;10(3):e0119144. doi: 10.1371/journal.pone.0119144 (PMC4367993; doi:10.1371/journal.pone.0119144)
Supplement: S4 Table — (DOCX) [file pone.0119144.s004.docx]

Table S4. PBDEs concentrations (pg/m^3^) in different particulate matter and gas phase in August, 2012.

|  | PM_2.5_ | gas | PM_2.5_ | gas | PM_10_ | gas | PM_10_ | gas | TSP | gas | TSP | gas |
| --- | --- | --- | --- | --- | --- | --- | --- | --- | --- | --- | --- | --- |
| BDE-28/33 | 0.65 | 2.14 | 0.51 | 2.47 | 0.21 | 8.89 | 0.15 | 1.88 | 0.35 | 1.95 | 0.75 | 3.24 |
| BDE-49 | - | 1.11 | - | 0.56 | 0.98 | 2.38 | 0.84 | 2.52 | 0.19 | 1.33 | 0.54 | 0.23 |
| BDE-47 | 3.24 | 9.99 | 0.94 | 8.57 | 0.65 | 9.68 | 0.87 | 10.3 | 2.56 | 15.2 | 2.88 | 12.3 |
| BDE-66 | 0.96 | 7.21 | 1.02 | 4.31 | 0.21 | 2.62 | 0.34 | 2.78 | 1.53 | 2.05 | 1.21 | 4.82 |
| BDE-100 | 0.74 | 3.57 | 0.65 | 4.25 | 0.63 | 0.84 | 0.52 | 1.47 | 0.15 | 3.11 | 0.41 | 2.14 |
| BDE-99 | 7.21 | 13.5 | 4.32 | 15.4 | 6.99 | 25.3 | 5.87 | 12.5 | 15.4 | 10.9 | 19.3 | 9.97 |
| BDE-154 | 0.98 | 2.13 | 1.14 | 0.55 | 0.75 | 2.58 | 1.02 | 1.65 | 1.02 | 0.09 | 1.56 | 0.78 |
| BDE-153 | 2.32 | 6.12 | 1.68 | 5.18 | 2.54 | 2.61 | 3.97 | 4.16 | 2.34 | 2.47 | 2.14 | 3.22 |
| BDE-138 | 2.13 | 2.41 | 2.41 | 1.98 | 3.53 | 0.11 | 2.98 | 0.24 | 5.98 | 0.13 | 3.88 | 0.08 |
| BDE-183 | 0.75 | 0.98 | 0.86 | 1.68 | 4.98 | - | 2.97 | - | 2.04 | 0.08 | 3.21 | 0.14 |
| BDE-196 | 1.51 | - | 1.18 | - | 3.87 | - | 2.54 | - | 3.58 | - | 4.99 | - |
| BDE-203 | 1.45 | - | 1.52 | - | 0.98 | - | 5.35 | - | 4.39 | - | 5.24 | - |
| BDE-208 | 1.94 | - | 2.65 | - | 4.35 | - | 6.35 | - | 7.98 | - | 8.54 | - |
| BDE-207 | 2.21 | - | 1.97 | - | 5.69 | - | 7.64 | - | 9.75 | - | 10.87 | - |
| BDE-206 | 2.98 | - | 3.42 | - | 9.71 | - | 11.3 | - | 14.1 | - | 13.4 | - |
| BDE-209 | 15.7 | - | 11.3 | - | 59.6 | - | 62.1 | - | 32.2 | - | 70.6 | - |
