# Supplementary material for: Polybrominated Diphenyl Ethers (PBDEs) in PM2.5, PM10, TSP and Gas Phase in Office Environment in Shanghai, China: Occurrence and Human Exposure
Source: PLoS One. 2015 Mar 20;10(3):e0119144. doi: 10.1371/journal.pone.0119144 (PMC4367993; doi:10.1371/journal.pone.0119144)
Supplement: S5 Table — (DOCX) [file pone.0119144.s005.docx]

Table S5. PBDEs concentrations (pg/m^3^) in different particulate matter and gas phase in December, 2012

|  | PM_2.5_ | gas | PM_2.5_ | gas | PM_10_ | gas | PM_10_ | gas | TSP | gas | TSP | gas |
| --- | --- | --- | --- | --- | --- | --- | --- | --- | --- | --- | --- | --- |
| BDE-28/33 | 0.89 | - | 0.52 | - | 0.49 | 5.91 | 0.45 | 5.33 | 1.32 | 6.58 | 1.9 | 7.84 |
| BDE-49 | 0.41 | 0.52 | 0.45 | 0.21 | 0.32 | 1.65 | 0.34 | 0.19 | 0.84 | 3.87 | 0.74 | 0.35 |
| BDE-47 | 1.39 | 21.5 | 3.25 | 16.4 | 1.98 | 25.5 | 2.31 | 19.7 | 1.74 | 37.8 | 1.98 | 19.7 |
| BDE-66 | 1.31 | - | 2.14 | - | 2.21 | 3.29 | 3.87 | 0.51 | 0.58 | 0.75 | 0.67 | 1.25 |
| BDE-100 | 0.97 | - | 0.84 | 0.97 | 0.87 | 3.51 | 1.15 | - | 0.31 | - | 0.87 | 1.88 |
| BDE-99 | 7.84 | 7.98 | 8.32 | 8.95 | 9.87 | 19.9 | 13.4 | 5.99 | 7.35 | 7.63 | 6.98 | 18.7 |
| BDE-154 | 2.14 | 1.31 | 2.98 | 2.03 | 1.59 | 1.41 | 3.12 | 2.95 | 1.98 | 3.02 | 2.21 | 1.04 |
| BDE-153 | 3.12 | 0.99 | 2.21 | 1.02 | 4.21 | 3.96 | 5.38 | 5.97 | 4.31 | 1.55 | 5.14 | 5.04 |
| BDE-138 | 4.21 | 1.79 | 3.86 | - | 3.21 | 0.83 | 7.98 | - | 4.32 | 3.31 | 4.87 | - |
| BDE-183 | 2.12 | - | 1.43 | - | 3.54 | 0.86 | 2.76 | 0.21 | 2.98 | 2.41 | 3.54 | 0.35 |
| BDE-196 | 1.98 | - | 1.53 | - | 2.98 | - | 3.14 | - | 5.97 | - | 4.03 | - |
| BDE-203 | 1.87 | - | 1.98 | - | 7.68 | - | 5.24 | - | 6.58 | - | 7.58 | - |
| BDE-208 | 3.98 | - | 3.54 | - | 10.3 | - | 12.8 | - | 7.32 | - | 18.9 | - |
| BDE-207 | 2.41 | - | 3.12 | - | 18.3 | - | 10.3 | - | 9.87 | - | 14.6 | - |
| BDE-206 | 3.65 | - | 3.89 | - | 7.52 | - | 13.97 | - | 15.9 | - | 13.8 | - |
| BDE-209 | 12.6 | - | 15.4 | - | 54.7 | - | 43.1 | - | 55.9 | - | 80.3 | - |
