# Supplementary material for: Polybrominated Diphenyl Ethers (PBDEs) in PM2.5, PM10, TSP and Gas Phase in Office Environment in Shanghai, China: Occurrence and Human Exposure
Source: PLoS One. 2015 Mar 20;10(3):e0119144. doi: 10.1371/journal.pone.0119144 (PMC4367993; doi:10.1371/journal.pone.0119144)
Supplement: S6 Table — (DOCX) [file pone.0119144.s006.docx]

Table S6. PBDEs concentrations (pg/m^3^) in different particulate matter and gas phase in January, 2013.

|  | PM_2.5_ | gas | PM_2.5_ | gas | PM_10_ | gas | PM_10_ | gas | TSP | gas | TSP | gas |
| --- | --- | --- | --- | --- | --- | --- | --- | --- | --- | --- | --- | --- |
| BDE-28/33 | 0.39 | 6.54 | - | 4.77 | 0.53 | 5.32 | 0.97 | 7.66 | 0.79 | 8.54 | 0.85 | 6.97 |
| BDE-49 | 0.41 | 1.01 | - | 3.52 | 0.51 | 0.58 | 0.43 | 0.44 | 0.54 | 1.13 | 0.61 | 3.87 |
| BDE-47 | 1.98 | 29.4 | 2.38 | 29.8 | 2.01 | 9.76 | 2.98 | 19.4 | 2.32 | 27.3 | 3.21 | 27.4 |
| BDE-66 | 0.76 | 5.98 | 0.86 | 4.73 | 1.79 | 1.94 | 1.52 | 4.05 | 1.95 | 5.77 | 2.54 | 6.24 |
| BDE-100 | 1.23 | 6.79 | 0.91 | 7.83 | 1.49 | 0.99 | 0.98 | 1.52 | 2.75 | 2.31 | 2.41 | 6.87 |
| BDE-99 | 7.81 | 35.9 | 9.18 | 30.1 | 9.51 | 15.7 | 12.7 | 25.3 | 13.4 | 30.4 | 18.8 | 31.1 |
| BDE-154 | 2.45 | 1.77 | 3.21 | 2.65 | 2.55 | - | 4.35 | - | 2.75 | - | 3.09 | 3.11 |
| BDE-153 | 3.69 | 6.12 | 3.96 | 2.65 | 3.98 | 2.44 | 4.32 | 4.52 | 4.23 | 4.31 | 2.21 | 3.87 |
| BDE-138 | 5.88 | 1.65 | 4.58 | 1.41 | 6.52 | 2.42 | 6.38 | 1.67 | 6.95 | 0.07 | 7.96 | 0.03 |
| BDE-183 | 3.01 | 1.54 | 2.45 | - | 3.62 | 0.75 | 4.53 | 0.52 | 5.62 | 1.65 | 4.21 | - |
| BDE-196 | 2.32 | - | 2.54 | - | 3.86 | - | 2.01 | - | 5.03 | - | 7.98 | - |
| BDE-203 | 2.05 | - | 2.41 | - | 6.34 | - | 4.05 | - | 8.95 | - | 9.54 | - |
| BDE-208 | 3.48 | - | 4.12 | - | 7.91 | - | 5.13 | - | 10.3 | - | 13.6 | - |
| BDE-207 | 2.65 | - | 2.87 | - | 9.88 | - | 7.92 | - | 13.9 | - | 11.9 | - |
| BDE-206 | 5.21 | - | 6.98 | - | 11.3 | - | 19.74 | - | 16.9 | - | 18.7 | - |
| BDE-209 | 18.3 | - | 23.9 | - | 59.5 | - | 47.2 | - | 76.5 | - | 85.2 | - |
