# Supplementary material for: Polybrominated Diphenyl Ethers (PBDEs) in PM2.5, PM10, TSP and Gas Phase in Office Environment in Shanghai, China: Occurrence and Human Exposure
Source: PLoS One. 2015 Mar 20;10(3):e0119144. doi: 10.1371/journal.pone.0119144 (PMC4367993; doi:10.1371/journal.pone.0119144)
Supplement: S7 Table — (DOCX) [file pone.0119144.s007.docx]

Table S7. PBDEs concentrations (pg/m^3^) in different particulate matter and gas phase in February, 2013

|  | PM_2.5_ | gas | PM_2.5_ | gas | PM_10_ | gas | PM_10_ | gas | TSP | gas | TSP | gas |
| --- | --- | --- | --- | --- | --- | --- | --- | --- | --- | --- | --- | --- |
| BDE-28/33 | 0.8 | - | 1.5 | 0.65 | 1.21 | 6.88 | 1.98 | 8.47 | 0.99 | 13.4 | 0.53 | 9.41 |
| BDE-49 | 0.53 | 2.77 | 0.25 | 0.99 | 0.97 | 2.89 | 0.74 | 2.83 | 1.11 | 1.91 | 0.51 | 1.11 |
| BDE-47 | 3.16 | 33.2 | 2.86 | 11.5 | 3.87 | 29.8 | 3.21 | 31.7 | 2.74 | 35.1 | 1.39 | 14.9 |
| BDE-66 | 0.98 | 3.35 | 0.78 | 5.67 | 1.24 | 3.11 | 2.12 | 4.21 | 2.74 | 3.06 | 3.12 | 4.11 |
| BDE-100 | 0.85 | 3.49 | 0.65 | 3.35 | 1.23 | 5.46 | 1.52 | 8.97 | 4.31 | 4.37 | 4.51 | 5.01 |
| BDE-99 | 10.9 | 30.2 | 13.4 | 10.9 | 15.3 | 35.4 | 14.2 | 37.9 | 16.3 | 15.8 | 15.9 | 13.7 |
| BDE-154 | 1.02 | - | 1.19 | 0.94 | 3.12 | - | 5.14 | 2.97 | 4.12 | 0.54 | 5.32 | 1.99 |
| BDE-153 | 2.32 | 4.71 | 2.85 | 5.21 | 4.57 | 4.14 | 5.21 | 5.03 | 3.75 | 4.87 | 5.32 | 4.13 |
| BDE-138 | 4.92 | 0 | 4.21 | 0.54 | 5.47 | 0.14 | 6.88 | 1.32 | 8.95 | - | 6.25 | - |
| BDE-183 | 2.04 | 1.32 | 2.95 | 1.12 | 2.97 | 0.32 | 2.54 | 0.41 | 5.98 | 1.67 | 6.52 | 2.65 |
| BDE-196 | 1.98 | - | 1.87 | - | 7.63 | - | 4.32 | - | 6.87 | - | 5.87 | - |
| BDE-203 | 1.98 | - | 2.32 | - | 5.98 | - | 7.14 | - | 10.8 | - | 13.2 | - |
| BDE-208 | 8.98 | - | 6.32 | - | 6.21 | - | 8.24 | - | 15.6 | - | 6.52 | - |
| BDE-207 | 3.54 | - | 5.98 | - | 7.85 | - | 8.56 | - | 15.3 | - | 20.6 | - |
| BDE-206 | 7.21 | - | 5.14 | - | 21.3 | - | 11.3 | - | 20.9 | - | 21.3 | - |
| BDE-209 | 35.3 | - | 24.1 | - | 54.1 | - | 69.3 | - | 70.3 | - | 89.3 | - |
